# Regulatory CD4^+^ T cells redirected against pathogenic CD8^+^ T cells protect NOD mice from development of autoimmune diabetes

**DOI:** 10.3389/fimmu.2024.1463971

**Published:** 2024-09-16

**Authors:** Dimitri Kakabadse, Dawei Chen, Sigal Fishman, Hadas Weinstein-Marom, Joanne Davies, Li Wen, Gideon Gross, F. Susan Wong

**Affiliations:** ^1^ Diabetes Research Group, Division of Infection and Immunity, Systems Immunity University Research Institute, Cardiff University School of Medicine, Cardiff University, Cardiff, United Kingdom; ^2^ Laboratory of Immunology, MIGAL, Kiryat Shmona, Israel; ^3^ Department of Biotechnology, Tel-Hai College, Kiryat Shmona, Israel; ^4^ Section of Endocrinology, Internal Medicine, School of Medicine, Yale University, New Haven, CT, United States

**Keywords:** NOD mice, type 1 diabetes, CD8 + T cells, CAR (chimeric antigen receptor) Treg cell therapy, antigen-specific regulatory T cells

## Abstract

**Introduction:**

In this study, we report a novel therapeutic approach redirecting antigen-specific CD4^+^ T cells recognizing a hybrid insulin peptide (BDC2.5 T cell receptor (TCR) transgenic CD4^+^ T cells) to attract and suppress islet-specific CD8^+^ T cells T cells in the non-obese diabetic (NOD) mouse model, and prevent the development of autoimmune diabetes.

**Methods:**

Purified BDC2.5 CD4^+^ T cells were induced to differentiate into regulatory T cells (Tregs). The Tregs were then electroporated with mRNA encoding chimeric human β_2_ microglobulin (hβ_2_m) covalently linked to insulin B chain amino acids 15-23 (designated INS-eTreg) or islet-specific glucose-6-phosphatase related protein (IGRP) peptide 206-214 (designated IGRP-eTreg). Immunoregulatory functions of these engineered regulatory T cells (eTregs) were tested by *in vitro* assays and *in vivo* co-transfer experiments with β-cell-antigen-specific CD8^+^ T cells in NOD.Scid mice or by adoptive transfer into young, pre-diabetic NOD mice.

**Results:**

These eTregs were phenotyped by flow cytometry, and shown to have high expression of FoxP3, as well as other markers of Treg function, including IL-10. They suppressed polyclonal CD4^+^ T cells and antigen-specific CD8^+^ T cells (recognizing insulin or IGRP), decreasing proliferation and increasing exhaustion and regulatory markers *in vitro*. *In vivo*, eTregs reduced diabetes development in co-transfer experiments with pathogenic antigen-specific CD8^+^ T cells (INS-CD8^+^ or IGRP-CD8^+^ cells) into NOD.Scid mice. Finally, when the eTreg were injected into young NOD mice, they reduced insulitis and prevented spontaneous diabetes in the recipient mice.

**Conclusion:**

Our results suggest a novel therapeutic strategy to protect NOD mice by targeting antigen-specific cytotoxic CD8^+^ T cells, using redirected antigen-specific CD4^+^ Treg cells, to suppress autoimmune diabetes. This may suggest an innovative therapy for protection of people at risk of development of type 1 diabetes.

## 1 Introduction

Autoreactive CD8^+^ and CD4^+^ T lymphocytes, together with B lymphocytes, are important cellular components of the immune infiltrate into the pancreatic islets of Langerhans in Type 1 diabetes (T1D) contributing to the loss of insulin-producing beta (β) cells in the Non-Obese Diabetic (NOD) mouse model of Type 1 diabetes. CD8^+^ T cells recognizing islet autoantigens insulin and islet-specific glucose-6-phosphatase related protein (IGRP) have a direct cytotoxic effect on the islets (1). Although autoreactive CD4^+^ T cells can themselves have a pathogenic effect in NOD mice (2, 3), they are often thought to be necessary for CD8^+^ T cells to function, providing help, particularly the production of IL-2.

In recent years, it is clear that regulatory T cells (Tregs) play a pivotal role in Type 1 diabetes. The reports of changes in Treg number are not in agreement, but there is consensus that their function is reduced. Furthermore, there is also evidence that pathogenic cells may be more difficult to regulate in Type 1 diabetes (4). Tregs suppress immune responses, aid in generating other regulatory lymphocytes, and may also prevent the onset of diabetes in the NOD mouse (5).

We have previously expressed a modified chimeric antigen receptor (CAR) on CD8^+^ T cells, inducing the gene-modified CD8^+^ cytotoxic T cells to target antigen-specific CD8^+^ T cells, which can protect NOD mice from the development of autoimmune diabetes (6–8). One of the targeted antigen-specific CD8^+^ T cells recognizes the insulin B chain peptide amino acids 15-23, and the G9Cα-/- T cell receptor (TCR) transgenic mouse, in which the majority of the CD8^+^ cells recognize this insulin B chain peptide, can be used to provide a source of insulin-specific CD8^+^ T cells for study (9), designated in this paper as INS-CD8^+^ T cells. These insulin-specific CD8^+^ T cells are important early in the pathogenic process and are amongst the earliest CD8^+^ T cells to be found in the pancreatic islets in NOD mice at 3-6 weeks (10, 11). We have also targeted CD8^+^ T cells that recognize the peptide 206-214 of islet-specific-glucose-6-phosphatase-related protein (IGRP) (12), in this paper designated IGRP-CD8^+^ T cells, and the NY8.3 TCR transgenic mouse can be used as a source of these cells. These IGRP-reactive CD8^+^ T cells start to rise a little later in the natural history of development of autoimmune diabetes at 7-10 weeks, and expand to become a predominant population of antigen-specific CD8^+^ T cells in the NOD mouse (10). Thus, engineering Treg expressing an MHC-peptide construct recognized by these pathogenic CD8^+^ T cells could be used as immunotherapy, and these engineered Treg cells (eTreg) will have the potential to target pathogenic antigen-specific T cells, regulating them specifically, with less likelihood of causing generalized immune suppression. Given the successful ability to express the constructs and selectively target antigen-specific cells (6–8), this raised the possibility that this strategy could be used to endow protective Tregs to regulate/remove pathogenic CD8^+^ T cells specifically.

Here we present a novel proof of concept that CD4^+^ Treg cells can be reprogrammed by electroporation with mRNA constructs encoding CD3-ζ-peptide/β_2_m (targeting CD8^+^ T cells) to specifically attract and interact with autoreactive T cells of defined antigen specificity. Recognition of target peptide presented by the eTreg cells reduced CD8^+^ T cell proliferation and induced regulatory/exhaustion markers on antigen-specific CD8^+^ T cells. Furthermore, co-adoptive transfer of these eTreg cells regulated antigen-specific CD8^+^ T cells, reduced insulitis and protected from diabetes in an adoptive transfer model of T1D, as well as the development of spontaneous diabetes in NOD mice.

## 2 Materials and methods

### 2.1 Mice

NOD mice, NY8.3 transgenic mice (12, 13), BDC2.5 TCR transgenic mice on the NOD genetic background (2), G9Cα-/- TCR transgenic mice (11), and NOD.Scid mice were bred in-house at Cardiff University. The mice were housed in specific pathogen-free facilities with a 12-hour light-dark cycle and provided *ad libitum* access to food and water. All experimental procedures involving animals were approved by the Cardiff University Animal Welfare and Ethics Review Committee and conducted under UK Home Office License approval, in accordance with the United Kingdom Animals (Scientific Procedures) Act, 1986, and associated guidelines.

### 2.2 mRNA *in vitro* transcription and delivery by electroporation

**Figure 1:**
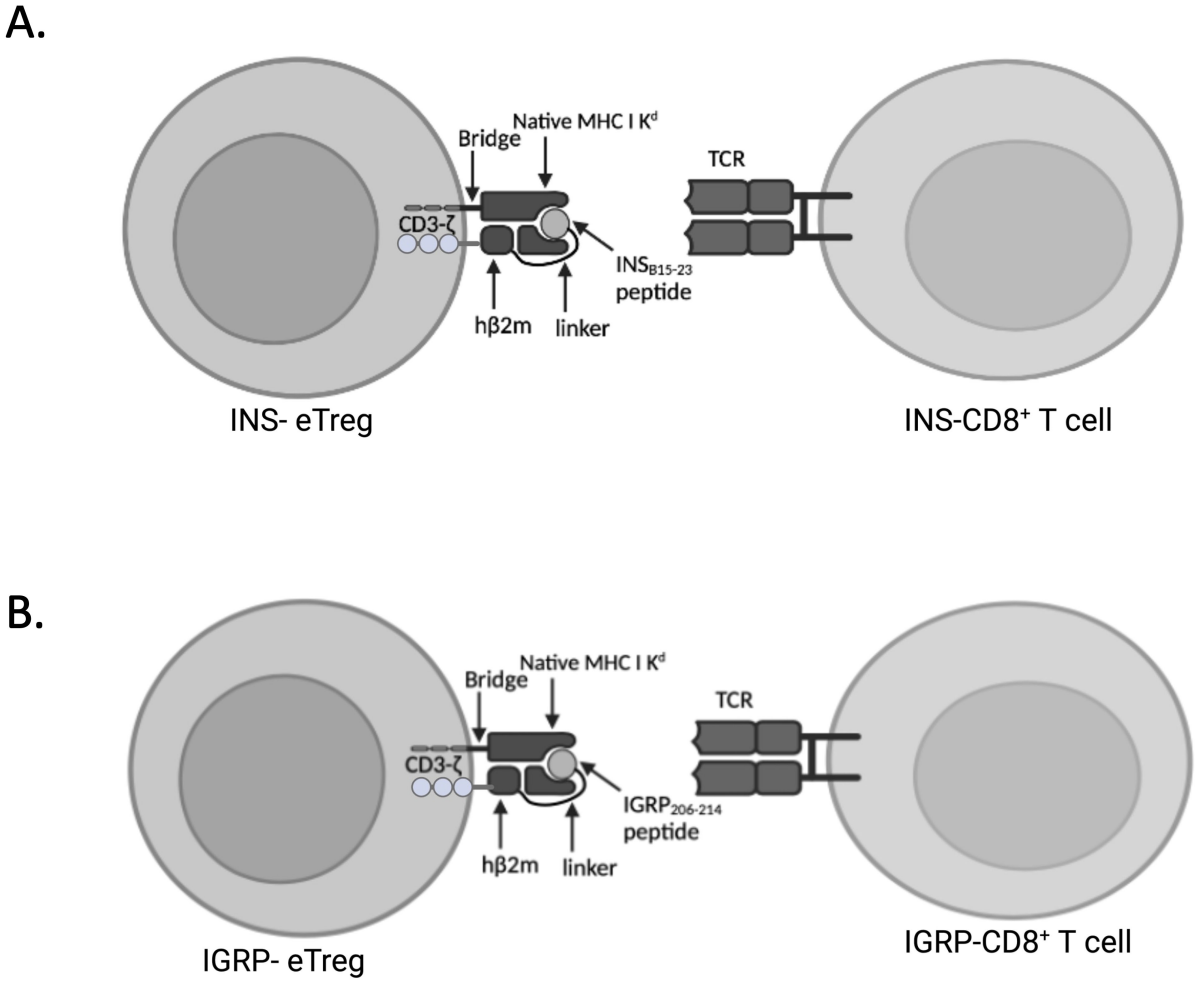
Expression of peptide linked to MHCI-K^d^ and intracellular CD3ζ eTreg constructs. **(A)** Schematic of the cell surface expression of mRNA encoding peptide/β_2_m/CD3-ζ, incorporating a modified InsB_15-23_ (LYLVCGERV) peptide designated INS-eTreg interacting with InsB_15-23_ specific G9Cα-/- CD8^+^ T cells designated INS-CD8^+^ T cells. **(B)** Schematic of the cell surface expression of mRNA encoding peptide/β_2_m/CD3-ζ, incorporating IGRP_206–214_ (VYLKTNVFL) peptide designated IGRP-eTreg interacting with IGRP_206-214_ specific NY8.3 CD8^+^ T cells designated IGRP-CD8 T cells. Figure created with BioRender.com/MZ278V5E0Y.

For *in vitro* transcription of mRNA, we used DNA templates encoding peptide/β_2_m/CD3-ζ, incorporating a modified InsB_15-23_ (LYLVCGERV) peptide designated INS construct or IGRP_206-214_ (VYLKTNVFL), designated IGRP construct for generation of INS-eTregs and IGRP-eTregs respectively, shown schematically in **Figures 1A, B**. These use endogenous MHC-I heavy chains and the engineered constructs, which comprise the chimeric polypeptide chains expressed with endogenous MHC class I (8). Human β_2_m (hβ_2_m) was used in this study, as it associates efficiently with mouse MHC-I heavy chains and can be used for detecting expression of the resulting MHC-I complexes (8). The pGEMT vector (Promega, Madison, WI) was used for direct cloning of PCR products. Template DNA for *in vitro* transcription of mRNA was cloned into the pGEM4Z/GFP/A64 vector (8) and prepared as previously described (8). mRNA constructs were transcribed *in vitro* using the T7 mScript Standard mRNA Production System (Cell-Script, USA) to generate 5’-capped mRNA. Cells were washed and resuspended in Opti-MEM medium (Gibco) containing the appropriate amount of *in vitro*-transcribed mRNA (10-20 μg). Electroporation was performed using BTX Harvard Apparatus ECM830 (1 millisecond, 300 V) into the prepared BDC2.5 CD4^+^ Treg cells (see below) to generate engineered Treg, hereafter designated eTreg. eTreg cells were only used for experiments if > 50% of the cells expressed the constructs, and this was assessed by performing flow cytometry before any *in vitro* or *in vivo* study, as described below in the flow cytometry section.

### 2.3 Generation of CD4^+^ Treg cells

**Figure 2:**
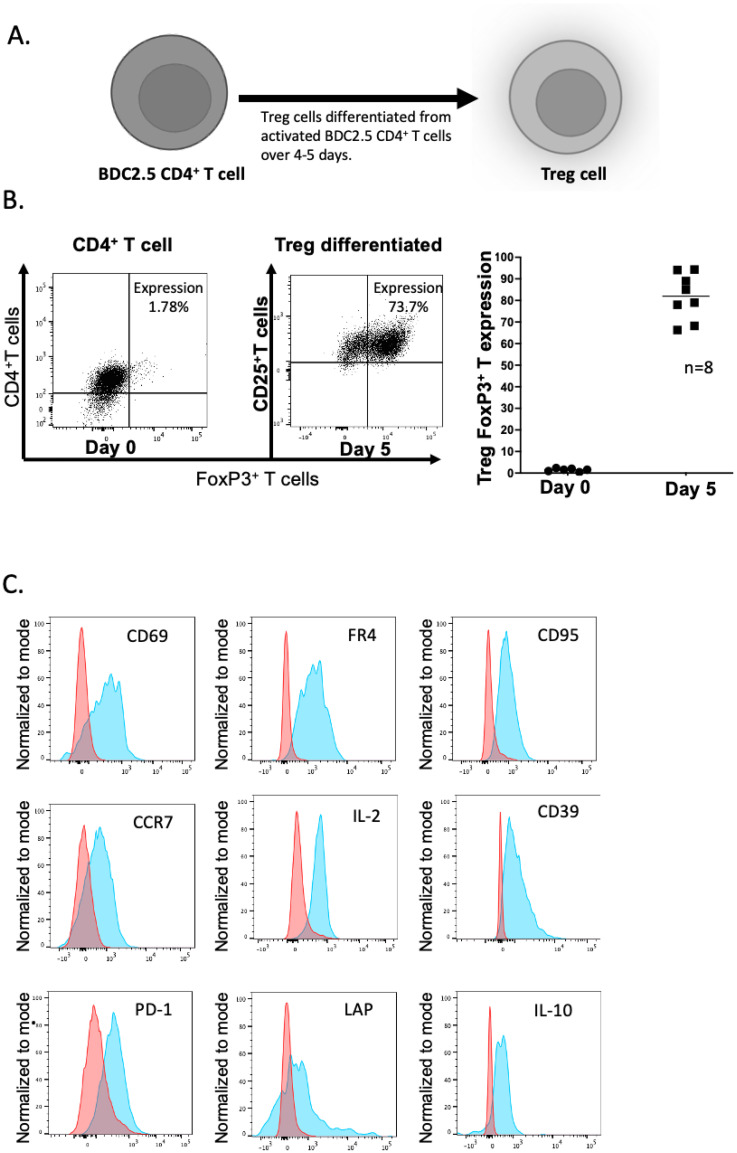
Differentiation of BDC2.5 hybrid insulin peptide-reactive BDC2.5 T cells to become Treg cells. **(A)** Differentiation process during 4-5 days of stimulation in culture. **(B)** Expression of FoxP3 and CD25 on the CD4^+^ T cells – flow cytometric plots shown on the left and graphical representation shown on the right. The plots are representative of 8 experiments. **(C)** Expression of CD69, FR4, CD95, CCR7, IL-2, CD39, PD1, LAP and IL-10. The plots show representative flow cytometry from 2-3 experiments. Figure created with BioRender.com/CG278SJQ4M.

Spleens from NOD BDC2.5 non-diabetic mice (6-10 weeks old) were homogenized, and erythrocytes were removed. CD4^+^ T cells were isolated using magnetic negative selection (MACS;Miltenyi Biotec, UK). The isolated T cells were stimulated for 96 hours with CellXVivo™ Mouse Treg Cell Differentiation Kit (R&D, CDK007) in RPMI Medium at 37°C, 5% CO_2_. This process yielded a highly enriched population of FoxP3^+^CD25^+^ Treg cells. Differentiation of BDC2.5 CD4^+^ T Cells into Treg cells was confirmed by FoxP3 and CD25 expression, measured by flow cytometry (**Figure 2**). All the Tregs used in this study were BDC2.5 CD4^+^ Treg.

### 2.4 Flow cytometry

For extracellular staining, cells were incubated with TruStain (Biolegend, UK) for 10 minutes at 4°C to block Fc receptors. Multi-parameter flow cytometry was carried out using the antibodies against the surface markers indicated as follows: CD8-FITC (53-6.7), CD4-AF700 (RM4-5), CD69-BV711 (H1.2F3), LAP-PerCP-Cy5.5 (TW7-16B4), PD1-BV605 (29F.1A12), Lag3-PE-Dazzle594 (C9B7W), CD304-PerCP-Cy5.5 (3E12), CD39-PE-Cy7 (Duha59), TIGIT-PE (1G9), CD95-FITC (SA367H8), CCR7-BV650 (4B12), (all from BioLegend), and FR4-BV605 (12A5), CXCR1-BV786 (5A12), TNF-α−PE-CF594 (3/23), CD49b-BV786 (HMα2) (all from BD Bioscience), and dead cells were excluded from the analysis using Live/Dead Viability Dye (Thermo Fisher).

For intracellular cytokine analysis, cells were treated with phorbol 12-myristate-13-acetate (PMA) (50 ng/ml), ionomycin (500 ng/ml), and monensin (3 μg/ml) (all from Sigma-Aldrich) for 3 hours. Fc receptors were blocked using TruStain, and after extracellular staining, cells were fixed using fixation/permeabilization according to the manufacturer’s instructions. Antibodies against intracellular markers, including FoxP3-APC (FJK-16s, eBioscience), CTLA4-PE (UC10-4B9, Thermo Fisher), IL-2 (JES6-5H4, BD Biosciences) IL10-APC (JES5-16E3, BD Biosciences), and IFNγ-PE-Cy7 (XMG1.2, BioLegend), or appropriate isotype controls, were used for staining.

For assessment of expression of hβ_2_m, transfected cells were first incubated in Fixation/Permeabilization buffer (eBioscience, 00-5523-00), followed by staining with anti-hβ_2_m FITC (clone 2M2, Thermo Fisher) together with antibodies against surface and intracellular markers. The other markers stained for included anti-FoxP3 Alexa Fluor 647 (clone 3G3, eBioscience). This protocol allowed for the assessment of genetic construct expression in the transfected eTregs.

Flow cytometry was performed on LSRFortessa (FACS Diva software), and analysis was performed using Flowjo software 10.8.1 (Treestar).

### 2.5 *In vivo* expression of transfected constructs in eTreg cells

BDC2.5 T regs cells were isolated, activated, and transfected with INS-construct or IGRP-construct mRNA and labelled with PKH lipophilic dye (Sigma Aldrich). As a control, BDC2.5 Treg cells were subjected to the electroporation procedure without added mRNA (mock-transfected) and labelled in the same way. The construct-transfected or mock-transfected eTregs (designated MOCK-eTreg) cells were adoptively transferred into 5-6-week-old NOD mice by intravenous injection into the tail vein. After 24, 48 hours and 5 days, mesenteric, and pancreatic lymph nodes as well as spleens were taken from the mice for measurement of the *in vivo* expression of the genetic constructs by staining for hβ_2_m in the INS-eTregs or IGRP-eTregs. In addition, other markers stained for included anti-FoxP3 Alexa Fluor 647 (clone 3G3, eBioscience), and cells were gated on PKH 26-positive cells. This protocol allowed for the assessment of genetic construct expression in the transfected eTregs.

### 2.6 CD8^+^ T cell death

The BDC2.5 CD4^+^ Treg cells were transfected with mRNA encoding INS or IGRP constructs to generate eTregs. CD8^+^ T cells were isolated from the spleens of 5- to 6-week-old G9Cα-/- or NY8.3 mice and separated by negative selection (MACS; Miltenyi Biotec). The purified CD8^+^ T cells were then activated and stimulation using plate-bound anti-CD3 (0.5 µg/ml) (clone 2C11, Bio X Cell) and anti-CD28 (5 µg/ml) (clone 37.51, Bio X Cell) antibodies in 96-well plates. Activated mRNA-transfected eTreg cells were incubated with the activated, purified INS- or IGRP-CD8^+^ T cells respectively for 72 hours at different cell ratios of Treg: CD8^+^ T cell ratio. Death was assessed in the live/dead gate on flow cytometric plots.

### 2.7 eTreg inhibition of polyclonal CD4^+^ T cells

Naive INS-CD8^+^ T cells were isolated and co-cultured with INS-eTreg at a 1:1 ratio. After 24 hours, the INS-eTregs were purified using a negative selection kit for CD4^+^ T cells (Miltenyi Biotec), removing the CD8^+^ T cells. Polyclonal CD4^+^ T cells were purified from splenocytes of NOD mice using negative selection (Miltenyi Biotec) and labelled with CellTrace Violet (CTV) (Thermo Fisher). These labelled polyclonal splenic CD4^+^ T cells were then incubated with the INS-eTreg and stimulated by anti-CD3/CD28 Dynabeads according to the manufacturer’s instructions (Thermo Fisher) at different ratios of INS-eTreg to polyclonal CD4^+^ T cells, maintaining the number of polyclonal CD4^+^ T cells constant (15x10^3^ cells), in 96-well flat-bottomed plates. The cells were cultured in RPMI complete medium at 37°C, 5% CO_2_ for 72 hours, and the inhibitory effect of the INS-eTreg on proliferation of the CTV-labelled polyclonal CD4^+^ T cells was investigated by assessing the dilution of CTV using flow cytometry.

### 2.8 e-Treg inhibition of antigen-specific CD8^+^ T cell proliferation

INS-CD8^+^ T cells were isolated, labelled with Carboxyfluorescein succinimidyl ester (CFSE) (Thermo Fisher) and co-cultured with INS-eTreg, stimulated by anti-CD3/CD28 Dynabeads, according to the manufacturer’s instructions (Thermo Fisher), for 72 hours at different cell ratios. Proliferation of the INS-CD8^+^ T cells was assessed by dilution of CFSE using flow cytometry.

### 2.9 *In vivo* adoptive transfer to NOD.Scid mice

BDC2.5 Treg cells were generated and transfected with the either INS or IGRP constructs. These INS-eTreg cells (10^7^) or IGRP-eTreg cells (10^7^) were then co-transferred with 10^7^ INS-CD8^+^ or IGRP-CD8^+^ T cells respectively into 5-6-week-old female NOD.scid mouse recipients by intravenous injection into the tail vein. In this experiment, as irrelevant peptide transfected controls, we also used cotransfer of INS-CD8^+^ T cells with IGRP-eTreg and IGRP-CD8^+^ T cells with INS-eTreg. Mice were monitored daily for glycosuria using Diastix strips (Bayer Diastix). Diabetes was confirmed by blood glucose levels >13.9 mmol/l following two consecutive positive readings. eTreg were only used for the experiments if >50% of eTreg expressed the construct, as assessed by staining for hβ2m.

### 2.10 *In vivo* adoptive transfer of eTreg to NOD mice for investigation of protection from diabetes

BDC2.5 Treg cells were generated and transfected with either INS or IGRP constructs. Ten million INS-eTregs were transferred into female NOD mice at 5-6 weeks of age, and a further ten million INS-eTreg cells transferred one week later (n=16). A separate set of female NOD mice of the same age were transferred with MOCK-eTregs as controls at the same times as the INS-eTregs (n=8). Similarly, in a separate cohort of mice, ten million IGRP-eTregs were transferred into NOD mice at 7-8 weeks of age, and a further ten million IGRP-eTreg cells transferred one week later (n=16). A control set of mice of the same age were transferred with MOCK-eTregs as controls at the same times as the IGRP-eTregs (n=8). Furthermore, we also have included data from untreated NOD mice from our NOD colony at the same time as the experimental quality controls for diabetes development, as we maintain a check on overall diabetes incidence routinely, but these were not part of the experiment. The mice were monitored weekly for glycosuria using Diastix strips (Bayer Diastix) until 35 weeks of age or until diabetes developed, confirmed by blood glucose levels >13.9 mmol/l, following two consecutive positive readings. eTreg were only used for the experiments if >50% of eTreg expressed the construct as measured by staining for hβ2m, as described above.

### 2.11 Histology

The pancreas was fixed in paraformaldehyde-lysine-periodate buffer overnight, infused with 10% sucrose in phosphate buffer, followed by 20% sucrose, as described previously (6). The pancreas was then embedded in OCT and snap-frozen for immunohistochemistry. Frozen sections, 10 μm thick, were stained with rat-anti-mouse CD4-biotin (clone RM4-5, Thermo Fisher), CD8-biotin (clone 53.6.7, Thermo Fisher), B220-biotin (clone RA3-6B2, Thermo Fisher), and anti-insulin-biotin (ICBTACLS) antibodies and detected with streptavidin-alkaline phosphatase and a Vector Red AP substrate kit (Vector Laboratories, Peterborough, UK), followed by counterstaining with hematoxylin. Insulitis was assessed in 4-5 mice per group, and 100-150 islets were scored. Scoring for insulitis is shown in the figure legend.

### 2.12 Statistical analysis

Unpaired Student’s t-tests were used to compare data between groups, or 2-way ANOVA when multiple group comparisons were done. The Chi-square test was used for the analysis of insulitis. Log-rank analysis was carried out for the adoptive transfer experiments. A p-value of less than 0.05 was considered statistically significant. All statistical analysis was carried out using Graphpad Prism v 10.1.1.

## 3 Results

### 3.1 Generation of islet-specific regulatory BDC2.5 CD4^+^ T cells

BDC2.5 TCR transgenic CD4^+^ T cells express the BDC2.5 TCR which recognizes an insulin-chromogranin A hybrid peptide, expressed in the pancreatic islets of NOD mice (14). These cells very effectively target the islets through recognition of this endogenous peptide, but the NOD BDC2.5 TCR transgenic mice do not themselves develop spontaneous diabetes, as endogenous Treg cells prevent this from occurring (15). Thus, we chose to use the BDC2.5 TCR transgenic CD4^+^ T cells, in order to engineer Tregs *in vitro* to endow these cells with the ability to target the pancreatic lymph nodes and islets by virtue of their TCR. The differentiation process was carried out over 4-5 days (illustrated in **Figure 2A**), yielding a population of Tregs with a high expression of FoxP3 and CD25 (**Figure 2B**) and other markers including the activation markers CD69, FR4, CD95, CCR7 and IL-2 (**Figure 2C**). We also stained for other markers previously demonstrated on Treg including CD39, PD-1, LAP, and IL-10 (**Figure 2C**); the expression of other regulatory markers including LAG3, TIGIT, CTLA-4 and CD304 was very low (data not shown).

### 3.2 Expression of the CD3ζ-INS and CD3ζ-IGRP constructs in regulatory BDC2.5 CD4^+^ T cells (eTreg) *in vitro* and *in vivo*

**Figure 3:**
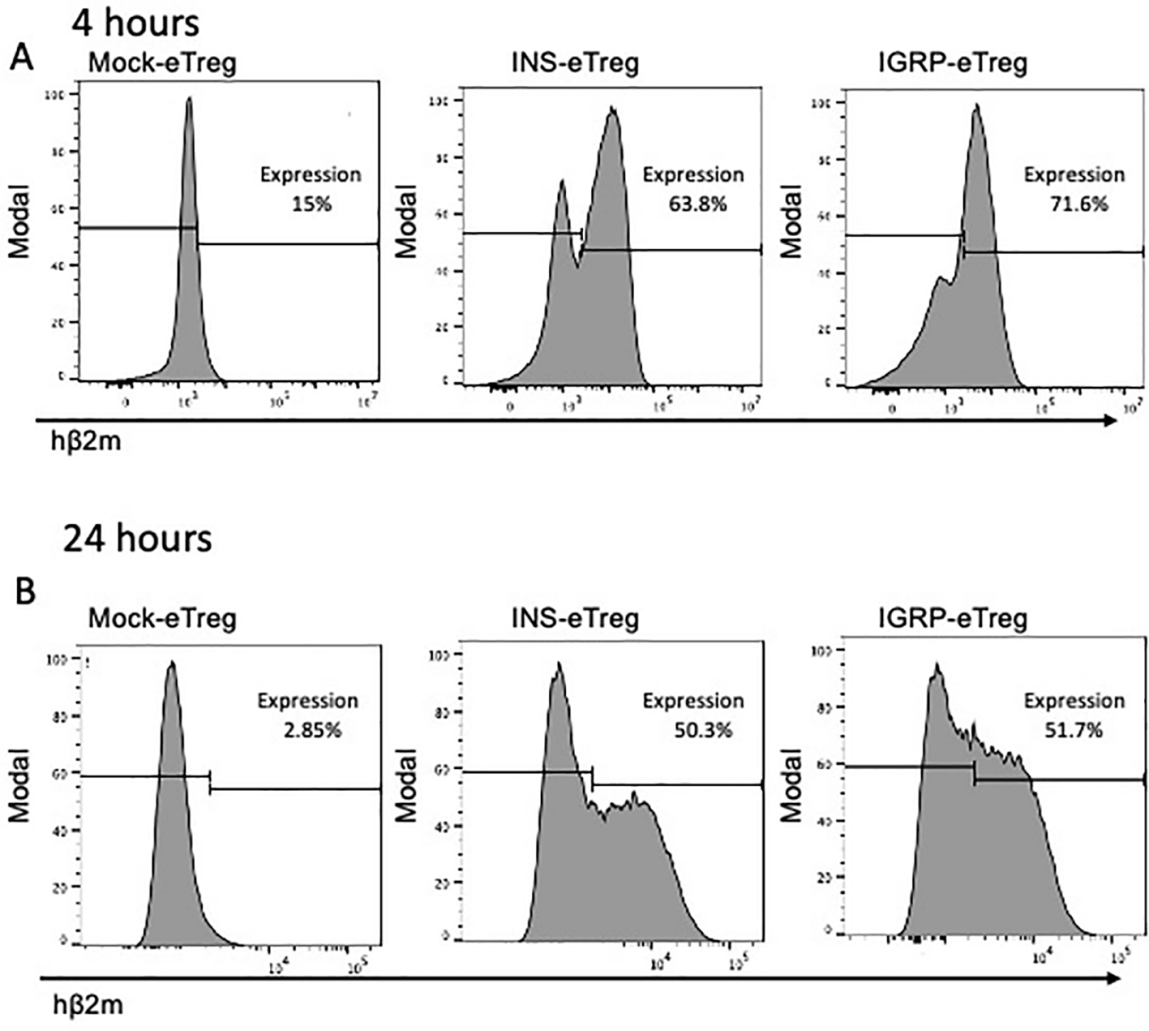
Expression of the INS and IGRP constructs on eTreg cells after transfection *in vitro*. Expression of transfected INS and IGRP constructs, as shown by staining for hβ2m on **(A)** eTregs at 4 hours and **(B)** eTregs at 24 hours *in vitro*. Representative of duplicates from 3 experiments.

To redirect the BDC2.5 TCR transgenic CD4^+^ Treg such that they would interact specifically with antigen-specific pathogenic CD8^+^ T cells, the BDC2.5 TCR transgenic CD4^+^ Treg were electroporated with the INS construct or the IGRP construct. Hereafter, the cells will be designated as INS-eTreg or IGRP-eTreg. Expression of these mRNA constructs was assessed, *in vitro*, 4 and 24 hours after transfection (**Figure 3**), by staining with anti-hβ_2_m. A mean of 67.5% (range 57.8-81%) of the INS-eTreg cells and 80.5% (range 71.6-88%) IGRP-eTreg expressed the constructs at 4 hours, decreasing to a mean of 53.3% INS-eTreg cells and 55.5% IGRP-eTreg cells at 24 hours (**Figure 3**). At 72 hours a mean of 14.1% INS-eTreg cells and 28.3% IGRP-eTreg cells expressed the constructs (data not shown).

**Figure 4:**
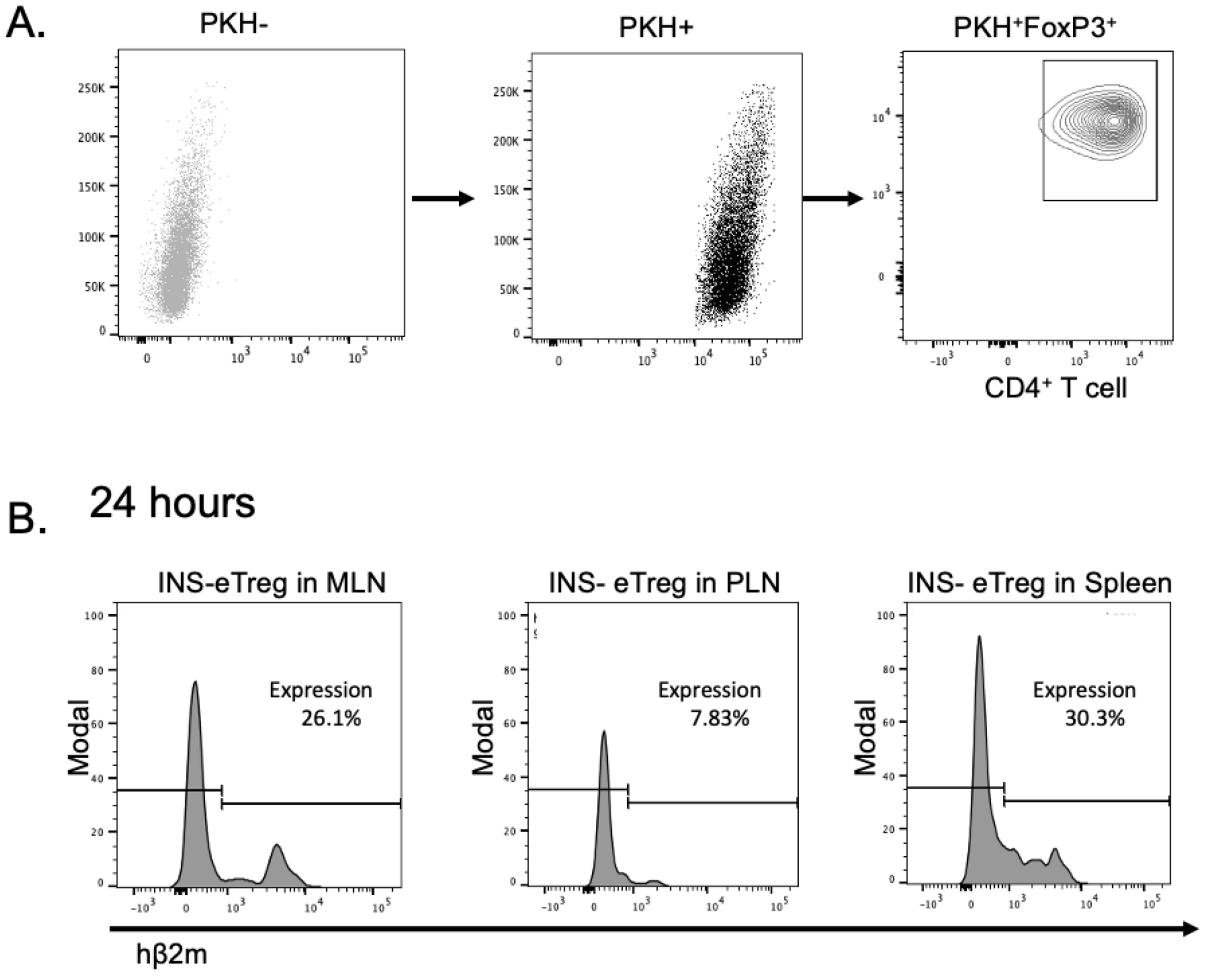
Expression of the INS and IGRP constructs on eTreg cells after transfection and adoptive transfer into NOD mice. **(A)** The INS transfected eTreg (shown as INS-eTreg) were stained with PKH lipophilic dye and expression of FoxP3 is shown in the INS-eTreg cells prior to intravenous injection into female NOD mice. **(B)** Expression of INS-eTreg construct in gated PKH^+^FoxP3^+^ CD4^+^ T cells, 24 hours after transfer into the NOD mice in mesenteric lymph nodes (MLN) (left panel), pancreatic lymph nodes (PLN) (middle panel) and spleen (right panel). Plots are representative of 4 mice from 2 experiments.

To investigate the expression of the constructs *in vivo*, we labelled the transfected eTreg that were positive for FoxP3, with the lipophilic dye PKH and 4 hours post labelling, we transferred the PHK-labelled eTreg, intravenously into NOD mice (**Figure 4A**). We tracked the presence of eTreg cells in the mesenteric lymph nodes (MLN), pancreatic lymph nodes (PLN) and spleen, 24 hours after transfer. The transfected mRNA constructs could still be clearly visualized up to 24 hours after adoptive transfer, as shown by expression of hβ_2_m in the PKH+ T cells (**Figure 4B**). Next, the cells were further tracked and mice were examined for presence of the Tregs, and the expression of the transfected constructs investigated at 5 and 8 days after transfer. Whereas the CD4^+^ PKH-labelled e-Treg cells, expressing FoxP3, could be detected in the lymph nodes at 5 and 8 days after transfer, the cells no longer expressed the constructs on their cell surface (data not shown).

### 3.3 Maintenance of CD4^+^ eTreg function

**Figure 5:**
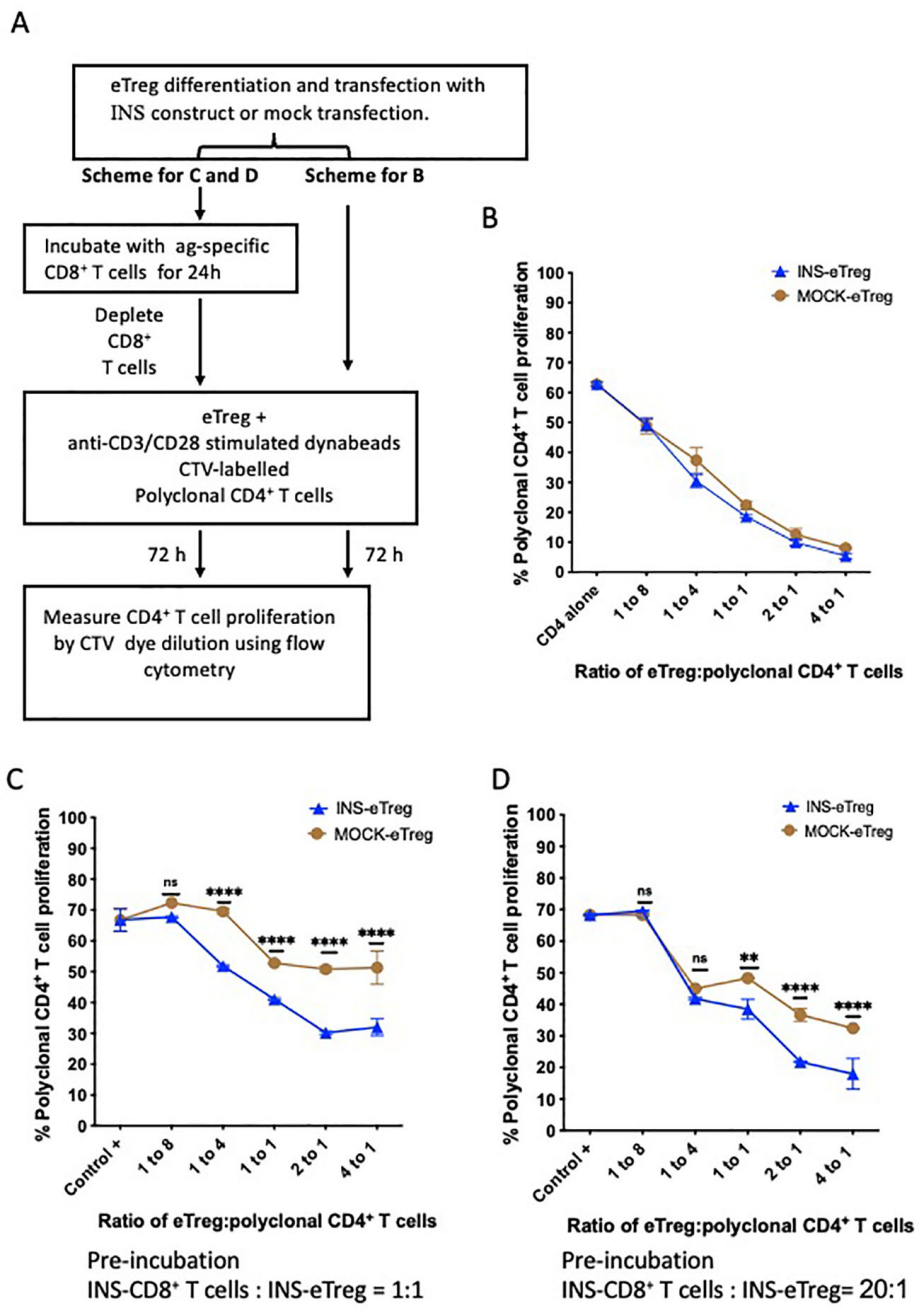
eTreg function after differentiation and transfection with INS construct and pre-incubation with INS-CD8^+^ T cells. **(A)** Experimental schematic demonstrating BDC2.5 CD4^+^ Treg differentiation over 5 days and INS construct transfection, followed by either direct incubation (no preincubation) with anti-CD3/CD28-stimulated CellTrace violet (CTV)-labelled polyclonal CD4^+^ T cells in a suppression assay, or pre-incubation with insulin-specific CD8^+^ T cells for 24 hours. Following pre-incubation, the INS-CD8^+^ T cells were then depleted and the INS**-**eTreg were then tested by incubation with anti-CD3/CD28-stimulated CTV-labelled polyclonal CD4^+^ T cells for 72 hours in the suppression assay. Proliferation of the CTV-labelled polyclonal CD4^+^ T cells was measured by dye dilution using flow cytometry. **(B)** Graph showing decrease in proliferation with increasing INS-eTreg or MOCK-eTreg to polyclonal CD4^+^ T cell ratio. Plot represents duplicate measurements in each of 4 experiments, with mean and SEM shown. There were no significant differences in proliferation at any of the ratios. **(C, D)** Graphs showing decrease in proliferation with increasing eTreg to polyclonal CD4^+^ T cell ratio, compared to MOCK-eTreg. The eTreg with CD8 T cell incubation at CD8^+^ T cell:eTreg ratio of 1:1 **(C)**, and 20:1 **(D)** are shown. Mean and standard deviation are shown. Graphs represent a combination of 2 separate experiments. **p<0.01; ****p<0.0001; ns, not statistically significant.

To investigate the regulatory function of the eTregs, these cells were cultured for 72 hours, in a Treg suppression assay with CTV-labelled polyclonal CD4^+^ T cell responders, which were purified from NOD splenocytes and stimulated with anti-CD3 and anti-CD28 Dynabeads (**Figure 5A**). With increasing proportions of eTregs to responder CTV-labelled polyclonal CD4^+^ T cells, the responder polyclonal CD4^+^ T cell proliferation decreased, as demonstrated by decrease in CTV dilution (**Figure 5B**). This assay tested general Treg function, and there was no difference overall in the general ability of eTregs to suppress CD4^+^ T cells, whether they were mock-transfected or transfected with the MHC-peptide complex.

To further test the regulatory function of the transfected eTregs, after encounter with their antigen-specific target T cells, the INS-eTregs were cultured with INS-CD8^+^ T cells as shown in the experimental schematic in **Figure 5A**. The eTreg were placed in culture with insulin peptide-specific INS-CD8^+^ T cells at different ratios of CD8^+^ T cell:eTreg, ranging from 1:1 to 20:1 to specifically prime the eTreg. After 24 hours, the eTregs were then separated from the priming CD8^+^ T cells by depleting the CD8^+^ T cells. As CD8^+^ T cells have cytotoxic function, we assessed the eTreg viability after the culture with INS-CD8^+^ T cells. We found that the incubation of eTreg with naive antigen-specific CD8^+^ T cells did not significantly affect the viability of the eTreg at different ratios of Treg: CD8 T cells (**Supplementary Figure S1**). Moreover, the eTreg were then used in a CD4^+^ Treg suppression assay culture for a further 72 hours, with anti-CD3- and anti-CD28-stimulated polyclonal CD4^+^ T cells, and we observed that the eTregs maintained, and enhanced their suppressive function toward polyclonal CD4^+^ T cells, after interaction with the CD8^+^ T cells, as shown for 2 different ratios of CD8:eTreg pre-culture (**Figures 5C, D**). Although mock-transfected eTreg increased suppressive activity, possibly related to the inflammatory cytokines produced from the CD8^+^ T cell incubation, it was clear that the antigen-specific eTreg suppressive function was more enhanced, and this effect was greater with the increased CD8^+^ T cell:eTreg ratio of 20:1 compared to 1:1. Thus, the eTregs maintained/enhanced their regulatory function after specific interaction with the antigen-specific CD8^+^ T cells.

### 3.4 Culture with eTreg cells increased CD8^+^ T cell death and reduced proliferation of CD8^+^ T cells

**Figure 6:**
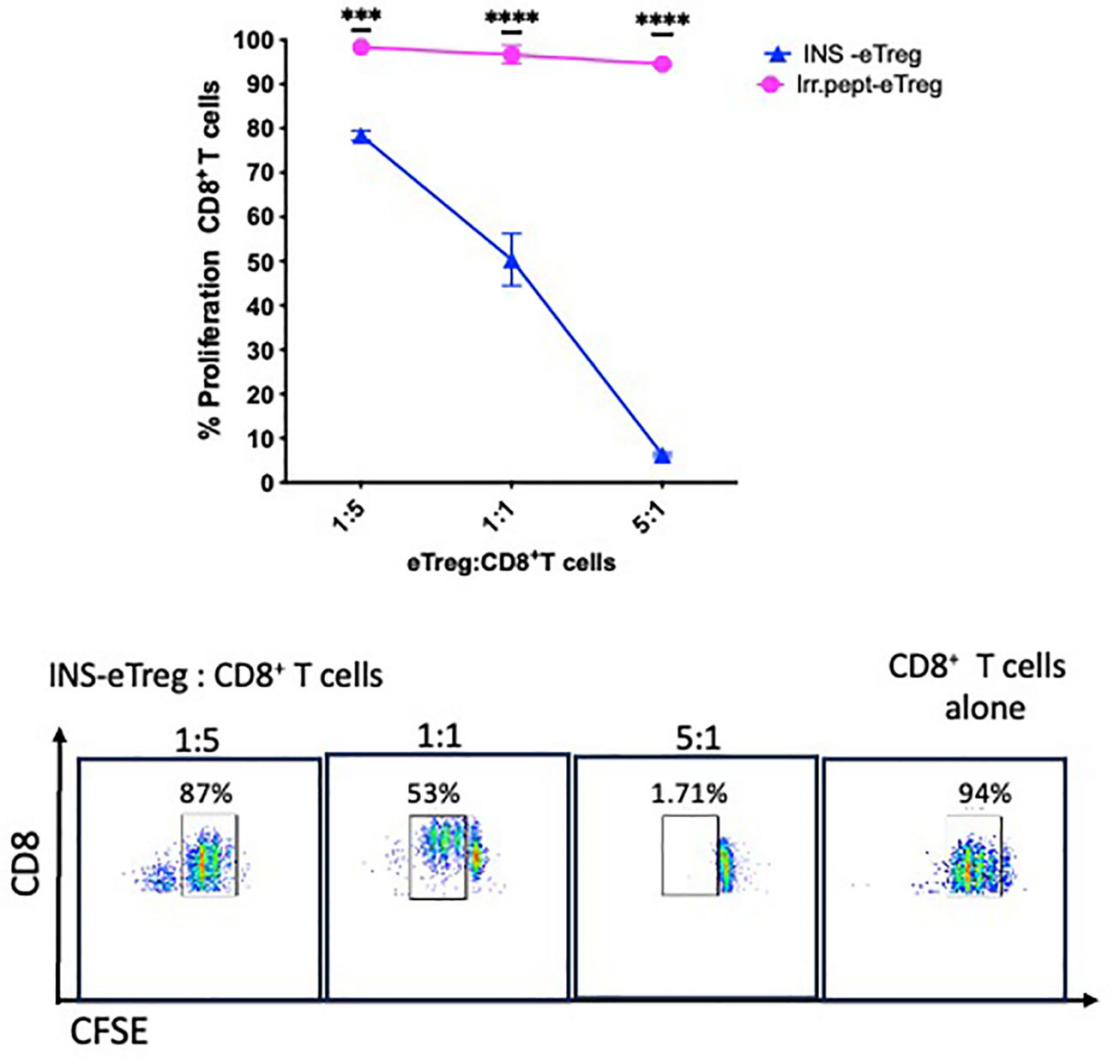
eTreg reduce proliferation of CD8^+^ T cells. INS-eTregs were incubated with CFSE-labelled INS-CD8^+^ T cells at different eTreg: CD8^+^ T cell ratios for 72 hours, and compared with incubation with irrelevant peptide-transfected eTregs (IrrPept-eTreg), shown in the graph (above), measuring proliferation by dilution of the intensity of CFSE. Plots demonstrating the dilution of the CFSE-labelled CD8^+^ T cells at the different INS eTreg:CD8^+^ T cell ratios are shown below. The graph showing mean and standard deviation is representative of 2 independent experiments, with 2-way ANOVA statistical analysis. ***p<0.001; ****p<0.0001; ns, not significant.

We have shown that the eTregs maintain polyclonal suppressive function after transfection with the specific MHC-peptide constructs, and next we investigated the specific effects of the eTregs on the antigen-specific CD8^+^ T cells. To do this, we first evaluated whether the antigen-specific eTreg induced death of the antigen-specific CD8^+^ T cells, and observed more loss of the antigen-specific CD8^+^ T cells after culture with the antigen-specific eTregs, compared with the cells incubated with MOCK-eTregs (**Supplementary Figure S2**). We then labeled the antigen-specific CD8^+^ T cells with CFSE and tested the CD8^+^ T cell proliferative responses by examining the dilution of the dye after incubation with the eTreg. We found that at a low ratio of CD4^+^ eTregs to CD8^+^ T cells, there was approximately 20% suppression, but with increased CD4^+^ eTregs to CD8^+^ T cell ratio, the CD8^+^ T cell proliferation was further reduced by 95% (**Figure 6**). Very little suppression of the antigen-specific CD8^+^ T cells (less than 10%) was seen in the presence of the eTregs transfected with an irrelevant peptide (IrrPept-eTreg).

### 3.5 Culture with the INS-eTreg cells altered the phenotype of CD8^+^ T cells, increasing the expression of regulatory markers

**Figure 7:**
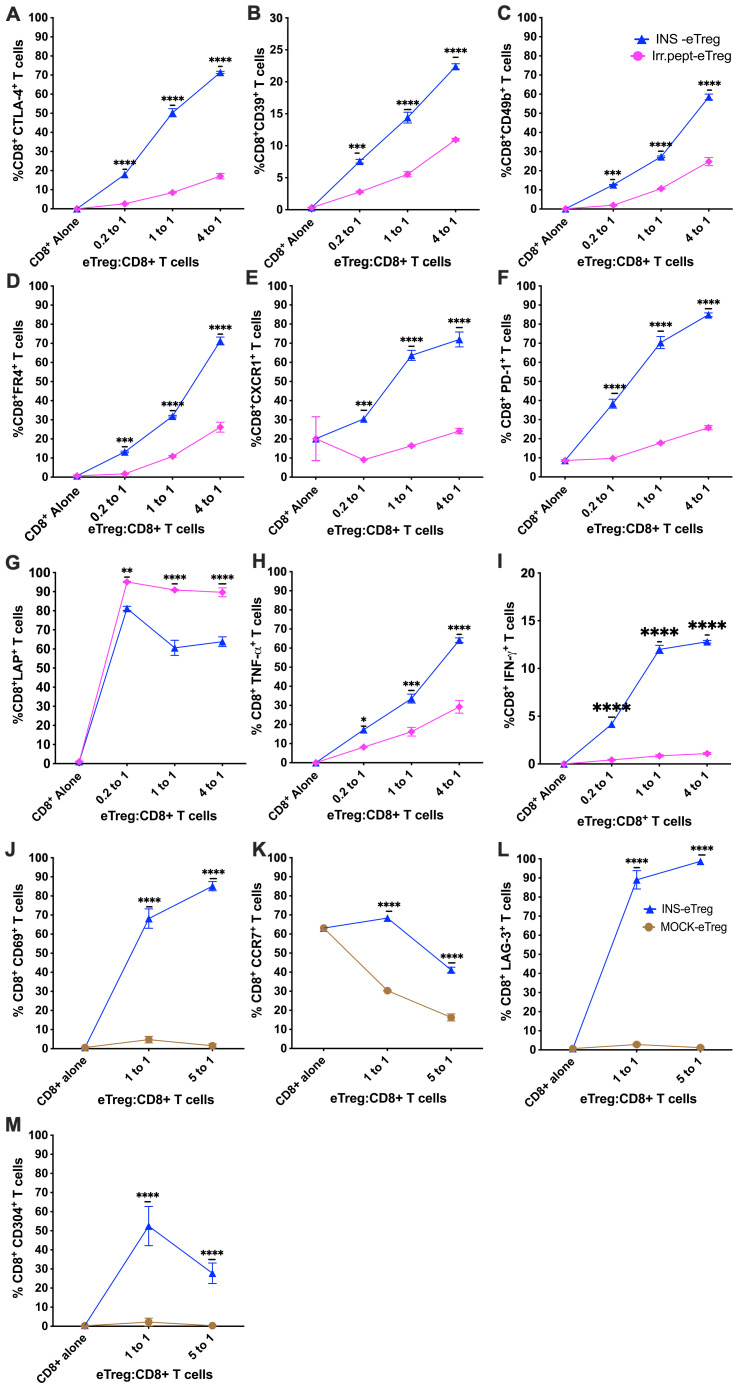
eTeg increase activation and exhaustion protein expression on CD8^+^ T cells. INS-eTreg cells were incubated with naïve INS-CD8^+^ T cells or IrrPept-eTreg or MOCK-eTreg cells for 72 hours. The CD8^+^ T cells were co-stained with antibodies against regulatory proteins CTLA-4 **(A)**, CD39 **(B)**, CD49b **(C)**, FR4 **(D)** migration marker CXCR1 **(E)**, markers of exhaustion PD1 **(F)**, as well as cytokines LAP **(G)**, TNFα **(H)**, IFNγ **(I)** and compared against culture with IrrPept-eTreg. N=8 from 3 independent experiments, with mean and SD shown and statistical analysis by 2-way ANOVA. Further tests were performed to test for CD69 **(J)** CCR7 **(K)**, LAG-3 **(L)** and CD304 **(M)** compared against culture with MOCK-eTreg. N=8 from 2 independent experiments. *p<0.05, **p<0.01, ***p<0.001, ****p<0.0001.

Having demonstrated that the eTregs suppressed the proliferation of naïve antigen-specific CD8^+^ T cells, we assessed the phenotype of the CD8^+^ T cells following culture with the antigen specific-eTregs and compared with either IrrPept-eTregs transfected with irrelevant peptide (**Figures 7A–I**) or MOCK-eTregs (**Figures 7J–M**). We found that there was upregulation of markers of exhaustion (PD1, FR4), as well as regulatory proteins (CD49b, CTLA-4, CD39, LAG3, CD304) activation markers (CD69), migration proteins (CXCR1, CCR7), and cytokines (TNFα, interferon-γ), although LAP was not increased.

### 3.6 Adoptive transfer of eTreg cells protected NOD.Scid mice from autoimmune diabetes induced by antigen-specific CD8^+^ T cells

**Figure 8:**
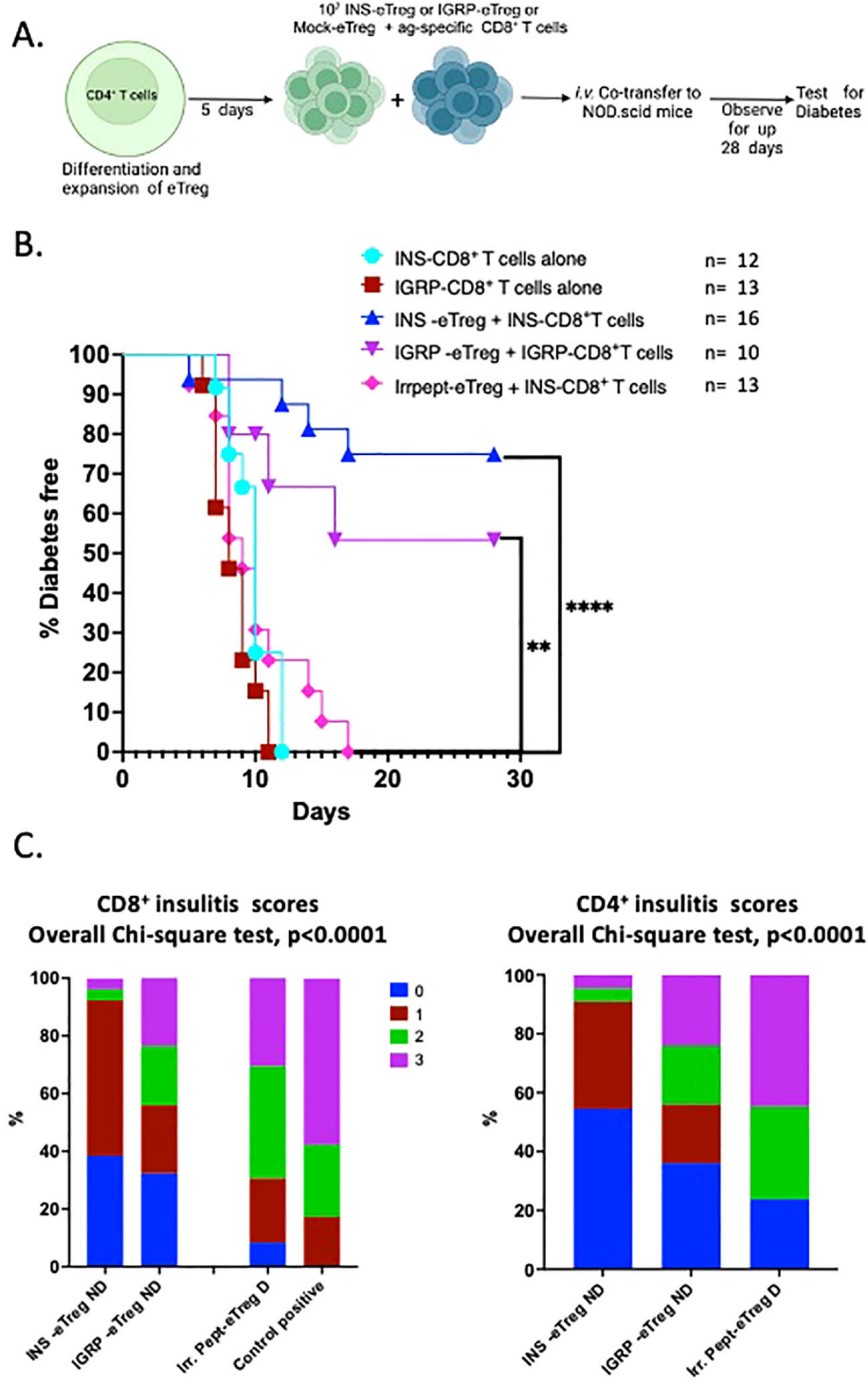
e-Tregs delay and protect against the development of T1D induced by adoptive transfer of pathogenic CD8^+^ T cells when co-transferred into NOD.Scid mice. **(A)** Schematic diagram illustrating the *in vivo* adoptive transfer. **(B)** Diabetes incidence in mice co-adoptively transferred at a 1:1 eTreg: CD8^+^ T cell ratio, with INS-eTreg transferred together with insulin-reactive CD8^+^ T cells or the IGRP-eTreg, transferred together with IGRP-CD8^+^ T cells or IrrPept-eTreg (5x10^6^ cells) together with INS- CD8^+^ T cells or IGRP-CD8^+^ T cells (5x10^6^ cells). As positive controls, INS-CD8^+^ T cells and IGRP-CD8^+^ T cells (5x10^6^ cells) were each transferred alone. Log rank test was performed. **p<0.01 ****p<0.0001. **(C)** Insulitis scores in recipient mice co-adoptively transferred at a 1:1 eTreg cell:CD8^+^ T cell ratio showing CD8^+^ T cell insulitis (left graph) and CD4^+^ T cell insulitis (right graph) in non-diabetic mice cotransferred with INS-eTreg + INS-CD8^+^ T cells, IGRP-eTreg + IGRP-CD8^+^ T cells, and IrrPept-eTreg co-transferred with INS-CD8^+^ T cells or IGRP-CD8^+^ T cells. The CD8^+^ T cell insulitis (left graph) with CD8^+^ T cells transferred alone is shown as positive control (left graph). Insulitis was scored from 3 mice per group, 22-38 islets with scoring as follow: 0=no insulitis, 1=peri-insulitis, 2=less than 50% infiltration, 3=greater than 50% infiltration. Data were analyzed by the Chi-square test. Figure created with BioRender.com/GB278SJSA2.

We had shown that eTregs suppressed both polyclonal CD4^+^ T cells and antigen-specific CD8^+^ T cells *in vitro*, and next the important question was whether eTregs suppressed the diabetogenic T cells *in vivo*. Thus, we tested the suppressive function of eTreg cells on pathogenic INS-CD8^+^ T cells and IGRP-CD8^+^ T cells in adoptive co-transfer into NOD.Scid mice. The experimental scheme is shown in **Figure 8A**. Activated splenic INS-CD8^+^ T cells, isolated from G9Cα^-/-^ mice, or activated IGRP-CD8^+^ T cells, isolated from NY8.3 mice were transferred into NOD.Scid mice. When the activated antigen-specific CD8^+^ T cells were adoptively co-transferred with either INS-eTregs together with INS-CD8^+^ T cells (1:1) or IGRP-eTregs together with IGRP- CD8^+^ T cells (1:1) diabetes development was significantly reduced, compared with 100% diabetes occurring in the absence of the eTregs (**Figure 8B**) (p<0.001). When the pathogenic CD8^+^ T cells were co-transferred with control eTregs that were transfected with construct linked with an irrelevant peptide, all the mice development diabetes, and no protection was seen. Comparing the histology of the non-diabetic mice co-transferred with the pathogenic CD8^+^ T cells, the overall level of infiltration of CD8^+^ T cells was less severe, with lower levels of both infiltrating CD8^+^ T cells as well as CD4^+^ T cells (**Figure 8C**).

### 3.7 Adoptive transfer of eTreg cells protected NOD mice from autoimmune diabetes

**Figure 9:**
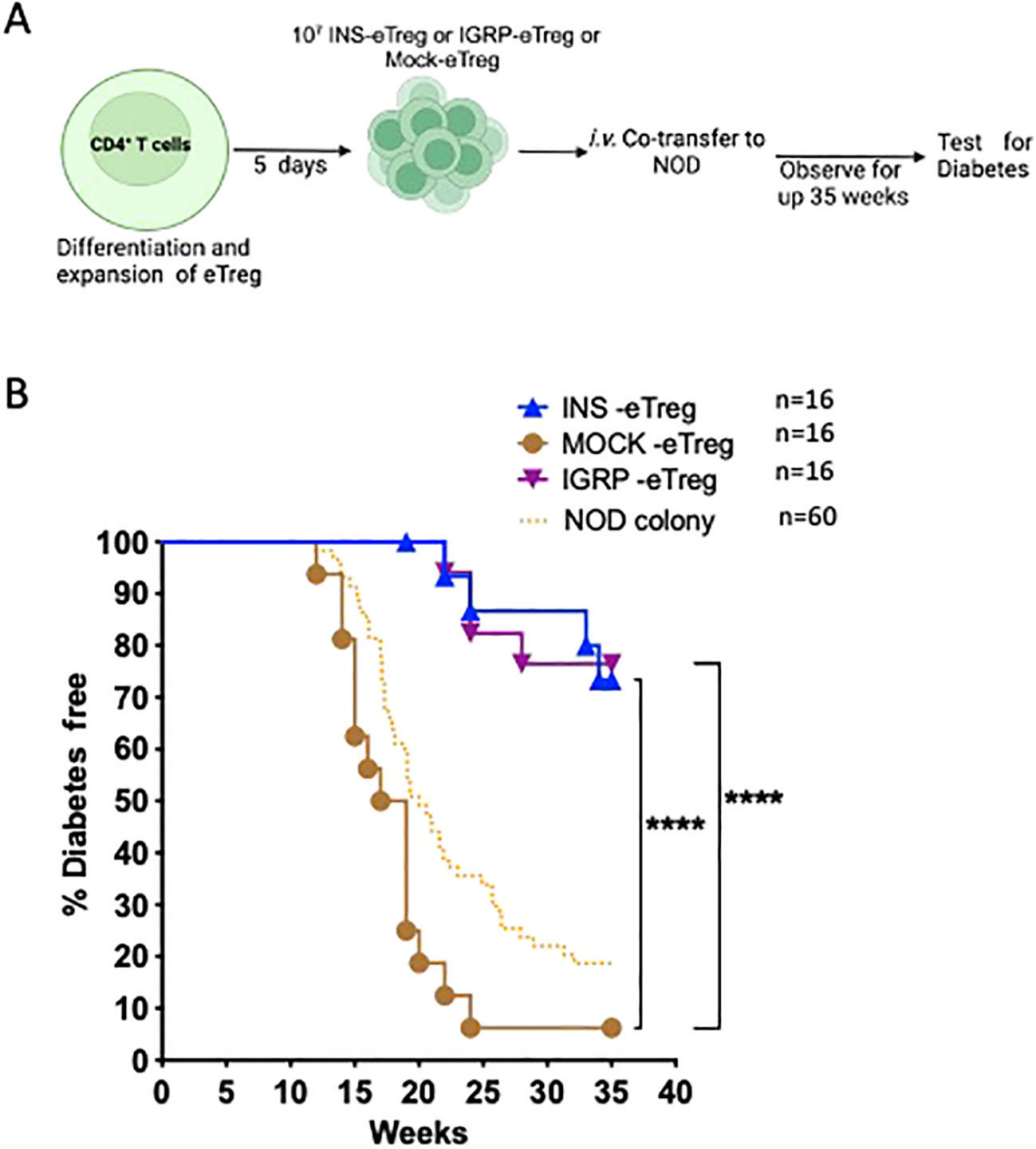
e-Tregs delay and protect against the development of T1D in NOD mice. **(A)** Schematic diagram illustrating the *in vivo* adoptive transfer. **(B)** Diabetes incidence in mice adoptively transferred with 2 iv injections of 10^7^ INS-eTreg cells, at 6 weeks and 7 weeks or IGRP-eTreg transferred at 8 weeks and 9 weeks. Control MOCK-eTreg were transferred into one group of mice at 6 and 7 weeks, and a separate group of mice at 8 and 9 weeks. The mice were observed for diabetes over 35 weeks and tested weekly for glycosuria after 11 weeks of age and diabetes diagnosed by a blood glucose measurement of >13.9 mmol/l. The incidence of diabetes in the NOD colony over the period of time that the experiments were ongoing is shown for quality control of the colony only. Log rank test was performed. ****p<0.0001. Figure created with BioRender.com/QC278SJU16.

Having shown that the eTregs were able to protect against adoptively-transferred CD8^+^ T cell-induced diabetes, we then tested eTreg effect on the incidence of spontaneous diabetes in NOD mice. We infused INS-eTreg cells into NOD mice at 5-6 weeks of age and repeated the infusion one week later again at 6-7 weeks of age. In a separate group of mice, the IGRP_-_eTregs were adoptively transferred into 7-8 week-old female NOD mice and this infusion was repeated one week later in 8-9 week old mice. These times were chosen related to the previously published reports that insulin-reactive CD8^+^ T cells appeared early (10, 11) whereas IGRP-CD8^+^ T cells occurred later (10). These mice were tested weekly for glycosuria and diabetes confirmed by blood glucose reading and the mice were terminated, either when they became diabetic, or at 35 weeks at the end of the experiments as shown in **Figure 9**.

### 3.8 NOD mice protected by eTreg transfer had reduced insulitis

**Figure 10:**
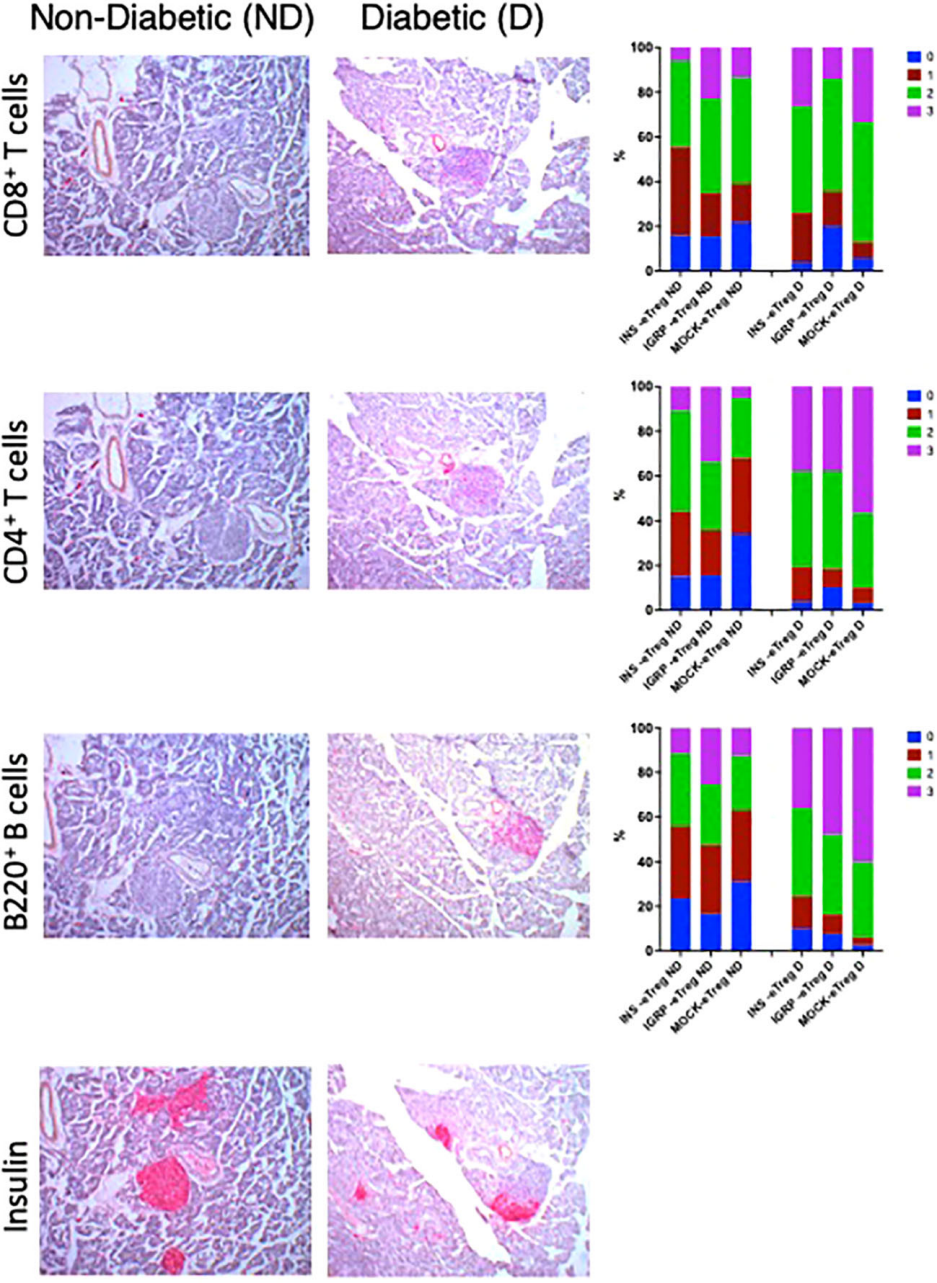
e-Tregs reduce insulitis and preserve insulin-containing islets. Insulitis scores in recipient mice from the experiment shown in **Figure 9**. Representative CD8^+^ T cell insulitis, CD4^+^ T cell insulitis, B cell insulitis, and insulin-stained islets in non-diabetic mice (left column) transferred with eTreg (twice), and were non-diabetic at 35 weeks of age, and diabetic mice (center column) transferred twice with MOCK-eTreg and became diabetic. The CD8^+^ T cell, CD4^+^ T cell and B cell insulitis scores are shown in the right column. Insulitis scores were as follow: 0=no insulitis, 1=peri-insulitis, 2=less than 50% infiltration, 3=greater than 50% infiltration. Data were analyzed by the Chi-square test and for CD8^+^ T cell, CD4^+^ T cell and B cell insulitis scores, p<0.0001.N: 4-5 mice for each group with 100-217 islets assessed.

We examined the insulitis in the pancreas samples of eTreg recipients, when the recipient mice were terminated (either when they developed diabetes or when non-diabetic at the end of the experiment at 35 weeks). We found reduced severity of insulitis in the protected mice (**Figure 10**), with less infiltration of CD8^+^ T cells, CD4^+^ T cells as well as B cells compared with those that developed diabetes. In contrast, the majority of those control recipients receiving the MOCK-eTreg cells became diabetic.

## 4 Discussion

In this study, we designed and tested a strategy to engineer antigen-specific eTreg cells that express chimeric antigen-MHC constructs which interact with 2 different pathogenic islet-autoantigen-specific CD8^+^ T cells (schematic in **Figure 1**). Using the strategy to convert CD4^+^ T cells that recognize a hybrid insulin peptide (BDC2.5 TCR transgenic cells) to Treg cells (**Figure 2**), we generated Tregs that expressed FoxP3 and various markers of induced Tregs, including the production of IL-10. These Tregs could traffic to PLN and islets, directed by virtue of their autoantigen specificity (hybrid insulin peptide) to the site where they could regulate autoantigen-specific CD8^+^ T cells. These BDC2.5 CD4^+^Treg were transfected with chimeric MHC-peptide complexes recognized by the pathogenic autoantigen-specific CD8^+^ T cells to generate the engineered Tregs (eTregs). We validated the expression of the constructs *in vitro* (**Figure 3**) and, *in vivo*, showing that the eTregs expressing the construct were present in MLN, PLN and spleen for at least 24 hours (**Figure 4**). The eTregs showed suppressive capacity, which was not only maintained but enhanced after pre-incubation with their cognate CD8^+^ T cells (**Figure 5**). The eTregs inhibited proliferation of the CD8^+^ T cells (**Figure 6**), and induced the CD8^+^ T cells to express activation, exhaustion, and regulatory T cell markers (**Figure 7**). They also promoted some cell death in the CD8^+^ T cells (**Supplementary Figure S2**). *In vivo*, the eTreg cells expressing the antigen-specific constructs recognized by pathogenic CD8^+^ T cells were able to protect against diabetes when co-adoptively transferred into immunodeficient NOD.Scid mice at a 1:1 eTreg to CD8^+^ T cell ratio (**Figure 8B**) and significantly decreased the severity of insulitis (**Figure 8C**). Most strikingly, the construct-expressing eTreg targeting the CD8^+^ T cells reduced the development of spontaneous autoimmune diabetes in NOD mice given only 2 doses at the pre-diabetic stage, in contrast to mock-transfected Tregs, which did not protect from diabetes when given with the same protocol in young mice (**Figure 9**). Insulitis was also shown to be reduced (**Figure 10**).

In T1D, endogenous Treg function is reduced, and this is one of the mechanisms involved in development of this autoimmune condition (16, 17). Thus, many studies have been directed towards increasing Tregs in order to rebalance towards immune tolerance. Adoptive Treg therapy has had some success in mice, and this has been enhanced by treatment using Treg expressing an antigen-specific TCR (18–20). The antigen specificity of the Tregs will direct them specifically to the tissue site of action. In our study, we took advantage of the islet antigen specificity of the BDC2.5 TCR transgenic CD4^+^ T cells recognizing hybrid insulin peptide to facilitate the targeting of the eTregs to the pancreatic lymph nodes/pancreatic islets, as these cells are known to traffic to pancreatic lymph nodes and to the pancreas. Although under other circumstances, these BDC2.5 T cells can be highly pathogenic, we converted them to become regulatory T cells with high expression of FoxP3. Furthermore, when endowed with the specific MHC class I-peptide CAR, the eTregs not only exerted regulatory activity over antigen-specific CD8^+^ T cells, but also showed increased suppressive activity toward bystander CD4^+^ T cells, after interaction with the antigen-specific CD8^+^ T cells (**Figure 10**). When transferred in naive form into non-immunosuppressed NOD mice, the TCR transgenic BDC2.5 T cells are not normally able to cause disease. Here, when BDC2.5 T cells were conditioned to become Tregs, mock-transfected and therefore without antigen-specific construct, at the early time points chosen for administration, they had no impact on the incidence of diabetes, inferring that they did not become additional pathogenic T cells, nor did they protect against diabetes. However, strikingly, the INS-construct and IGRP-construct expressing BDC2.5 eTregs protected against diabetes, in spite of the fact that the antigen-specific construct is only transiently expressed. This may suggest that the timing at which these antigen-specific eTregs were transferred is important, and that they are transferred at a time of increasing numbers of particular antigen-specific CD8^+^ T cells. In the future, we would aim to express the antigen-specific constructs for a longer period, perhaps by using viral transduction methods which would give increased longevity of construct expression.

Chimeric antigen receptor (CAR)-expressing T cells (CAR-T) have been much studied, especially in cancer immunotherapy. Studies have used different forms of CAR-Ts in diabetes models to alter a variety of cells, including CD8^+^ T cells (8, 21, 22), as well as regulatory T cells (23–25) and tested in NOD mouse model systems for protection from diabetes. Thus far, apart from our own earlier study (8), the majority of strategies adopted have targeted potentially pathogenic CD4^+^ T cells, with good targeting efficacy but varying effects on the development of autoimmune diabetes. Tenspolde and colleagues used Tregs generated by retroviral transduction of FoxP3 and then re-directed these cells with insulin-specific single chain antibody fragments (scFv) homing them towards beta cells of the islets in the pancreas (23). Their approach showed that the CAR-Tregs were stable, but did not protect NOD mice from diabetes development. Advancing this, Spanier and colleagues, generated CAR Tregs using scFv directed toward a peptide of the insulin B chain presented by the MHC class II I-A^g7^ found in the NOD mouse. When the engineered Treg cells were injected into NOD mice at 8-10 weeks of age, these CAR Tregs protected the mice from diabetes development and less than 50% of the mice had become diabetic compared with 80% of the non-Treg injected mice at 30 weeks of age (24). Obarorakpor and colleagues also targeted IA^g7^/insulin B9-23, and protected NOD.CD28-/- mice which develop accelerated diabetes (25). These strategies have all directed the Treg towards the islets and PLN, with specificities that could also regulate pathogenic CD4^+^ T cells. To our knowledge, our study is the first to endow antigen-specific Tregs with a modified CAR to specifically target early pathogenic CD8^+^ T cells that appear either early or later in disease development.

Our eTregs could potentially decrease the pathogenic effects of the CD8^+^ T cells in a number of ways. Firstly, coculture of the eTreg with their target antigen-specific CD8^+^ T cells caused increased CD8^+^ T cell death (**Supplementary Figure S2**). However, the cells were not eliminated, as we could detect antigen-specific CD8^+^ T cells in pancreatic lymph nodes and there were no clear changes in frequency of either insulin-specific or IGRP-specific CD8^+^ T cells, when assessed by antigen-specific tetramers (data not shown). Secondly, cytotoxic function of the CD8^+^ T cells, when in close proximity *in vitro* towards their antigenic targets was not affected (data not shown). The main effects of the eTregs on the CD8^+^ T cells were to decrease the proliferation of the antigen-specific cells (**Figure 6**), and increase exhaustion, with upregulation of PD-1 and other regulatory markers (**Figure 7**), which may have contributed to reduction in antigen-specific CD8^+^ T cell activity. Traffic to the islets was also reduced, as demonstrated by the reduction in the CD8^+^ T cell infiltration in the eTreg-transferred diabetes-protected mice (**Figure 10**). This concurs with the mechanism of action of CAR Tregs shown by others which decreased CD4^+^ T cell function (24, 25). Thus, we infer that protection occurred more likely in the pancreatic lymph nodes, with the reduced proliferation and traffic to the islets, and this reduction lessened the infiltration of CD8^+^ T cells in the islets, reducing diabetes.

Interest in therapeutic strategies in pre-clinical Type 1 diabetes has been increased with the advent of the FDA approval of Teplizumab, an anti-CD3 monoclonal antibody, in individuals who have been shown to have autoantibodies, and glycemic abnormalities on stimulation during a mixed meal tolerance test, but have not yet developed overt clinical diabetes (26, 27). Our strategy is designed for altering the immune processes at an early stage in the development of immune pathology, as we are aiming to target pathogenic CD8^+^ T cells at a time when these cells have been shown to be increasing in frequency (10), early on in the development of disease. Moreover, our strategy also halted the disease development after the early stage when pathogenic IGRP-reactive CD8^+^ T cells become predominant. It was not possible, related to the intensive resource required, to use larger numbers of experiments to test treatment later on in disease, and thus the timings were specifically chosen for this defined reason. At these time points, when the young NOD mice were transferred with mock-transfected Tregs, there was no significant effect on diabetes onset. In our study, we used unmanipulated NOD mice for our transfer experiments, and even though the MOCK-eTregs were themselves able to target the pancreas, by virtue of the expression of the BDC2.5 TCR, it appears that at these early time points, simply targeting the pancreas with Tregs may not sufficient. It should be noted that others who have transferred Tregs to test for reduction in diabetes in young mice have used different models, mostly either immunodeficient or accelerated models. Obarorakpor et al. (25), showed that their CAR Tregs, but not control Tregs (without the CAR construct), protected against diabetes when transferred to NOD.CD28-/- mice (deficient in Tregs and which develop accelerated diabetes) at a young age. Similarly, Tang and colleagues in an earlier publication also used NOD.CD28-/- mice, and showed that four Treg-treated NOD.CD28-/- mice were protected by the expanded BDC2.5 Tregs, but three untreated mice were not, when injected at the young age of 5 weeks (18). It is very clear that the environment early on in the pathogenic process in these Treg deficient NOD.CD28-/- mice is different from that later on.

We suggest that the timing of therapeutic administration is very important. Our aim in this paper was to show specificity in targeting antigen-specific CD8^+^T cells, particularly important in the earlier stages of diabetes, and therefore targeting them at this appropriate time. Without these early cells, the pathogenic process does not further develop, if there can be control at the earliest stages nearest to initiation. Once diabetes has already developed, in NOD mice transplanted with syngeneic islets, Tang and colleagues demonstrated that 2 of 5 diabetic NOD mice maintained remission of diabetes when injected after onset of diabetes, but 3 developed recurrent diabetes (18). They also showed that with a larger infusion of 10 million expanded Treg cells, 4 of 7 diabetic NOD mice had reversion to normoglycemia. This indeed shows the potential strength of an important strategy of using antigen-specific expanded T regs at the time of diabetes. In our strategy, where the Tregs have been administered a considerable time earlier, we would not expect that the Tregs would have the same effects as a large dose administered after diabetes. Therefore, we would suggest that at the early stage of administration of therapy, both targeting specificities are important, i.e., directing the eTreg to the site of pathogenic activity (as in the MOCK-eTregs), as well as the specific targeting to the antigen-specific CD8^+^ T cells.

In summary, our study utilizes the generation of CAR constructs, and has shown a proof-of-concept that targeting antigen-specific CD8^+^ T cells using islet antigen-specific Tregs has a long-lasting effect to protect NOD mice from development of autoimmune diabetes, when given in the early stages of pathogenesis of diabetes. We would anticipate refining this strategy, utilizing CAR constructs that incorporate costimulatory molecules which may increase efficacy, and potentially also using viral transformation which would increase longevity, but this would be a study for the future. This particular strategy is targeted at an early stage of diabetes pathogenesis, and thus, in consideration of adaptation of this strategy to humans would particularly require careful consideration of safety, as it would be important not to accelerate or precipitate clinical onset of diabetes if given before overt Stage 3 Type 1 diabetes. Consideration of the use of biomarkers for efficacy would be required, and the optimal timing of intervention would need to be considered, as it is clear that different protective effects may be obtained when Treg therapy is given at different time points in diabetes pathogenesis. Ultimately, the aim would be to translate this type of therapy to human type 1 diabetes.

## Data Availability

The original contributions presented in the study are included in the article/**Supplementary Material**. Further inquiries can be directed to the corresponding author.

## References

[1] Pugliese A. Autoreactive T cells in type 1 diabetes. J Clin Invest. (2017) 127:2881–91. doi: 10.1172/JCI94549 PMC5531393 28762987

[2] Katz JD Wang B Haskins K Benoist C Mathis D. Following a diabetogenic T cell from genesis through pathogenesis. Cell. (1993) 74:1089–100. doi: 10.1016/0092-8674(93)90730-E 8402882

[3] Mohan JF Calderon B Anderson MS Unanue ER. Pathogenic CD4(+) T cells recognizing an unstable peptide of insulin are directly recruited into islets bypassing local lymph nodes. J Exp Med. (2013) 210:2403–14. doi: 10.1084/jem.20130582 PMC3804950 24127484

[4] Schneider A Rieck M Sanda S Pihoker C Greenbaum C Buckner JH. The effector T cells of diabetic subjects are resistant to regulation via CD4+ FOXP3+ regulatory T cells. J Immunol. (2008) 181:7350–5. doi: 10.4049/jimmunol.181.10.7350 PMC2597079 18981158

[5] Chen Z Herman AE Matos M Mathis D Benoist C. Where CD4+CD25+ T reg cells impinge on autoimmune diabetes. J Exp Med. (2005) 202:1387–97. doi: 10.1084/jem.20051409 PMC2212985 16301745

[6] Scott GS Fishman S Khai Siew L Margalit A Chapman S Chervonsky AV. Immunotargeting of insulin reactive CD8 T cells to prevent diabetes. J Autoimmun. (2010) 35:390–7. doi: 10.1016/j.jaut.2010.08.005 20850948

[7] Scott GS Fishman S Margalit A Siew LK Chapman S Wen L. Developing a novel model system to target insulin-reactive CD8 T cells. Ann N Y Acad Sci. (2008) 1150:54–8. doi: 10.1196/annals.1447.040 19120267

[8] Fishman S Lewis MD Siew LK De Leenheer E Kakabadse D Davies J. Adoptive Transfer of mRNA-Transfected T Cells Redirected against Diabetogenic CD8 T Cells Can Prevent Diabetes. Mol Ther. (2017) 25:456–64. doi: 10.1016/j.ymthe.2016.12.007 PMC5368593 28109957

[9] Wong FS Siew LK Scott G Thomas IJ Chapman S Viret C. Activation of insulin-reactive CD8 T-cells for development of autoimmune diabetes. Diabetes. (2009) 58:1156–64. doi: 10.2337/db08-0800 PMC2671054 19208910

[10] Trudeau JD Kelly-Smith C Verchere CB Elliott JF Dutz JP Finegood DT. Prediction of spontaneous autoimmune diabetes in NOD mice by quantification of autoreactive T cells in peripheral blood. J Clin Invest. (2003) 111:217–23. doi: 10.1172/JCI200316409 PMC151866 12531877

[11] Wong FS Karttunen J Dumont C Wen L Visintin I Pilip IM. Identification of an MHC class I-restricted autoantigen in type 1 diabetes by screening an organ-specific cDNA library. Nat Med. (1999) 5:1026–31. doi: 10.1038/12465 10470079

[12] Lieberman SM Evans AM Han B Takaki T Vinnitskaya Y Caldwell JA. Identification of the beta cell antigen targeted by a prevalent population of pathogenic CD8+ T cells in autoimmune diabetes. Proc Natl Acad Sci U S A. (2003) 100:8384–8. doi: 10.1073/pnas.0932778100 PMC166238 12815107

[13] Verdaguer J Schmidt D Amrani A Anderson B Averill N Santamaria P. Spontaneous autoimmune diabetes in monoclonal T cell nonobese diabetic mice. J Exp Med. (1997) 186:1663–76. doi: 10.1084/jem.186.10.1663 PMC2199139 9362527

[14] Delong T Wiles TA Baker RL Bradley B Barbour G Reisdorph R. Pathogenic CD4 T cells in type 1 diabetes recognize epitopes formed by peptide fusion. Science. (2016) 351:711–4. doi: 10.1126/science.aad2791 PMC4884646 26912858

[15] Kanagawa O Militech A Vaupel BA. Regulation of diabetes development by regulatory T cells in pancreatic islet antigen-specific TCR transgenic nonobese diabetic mice. J Immunol. (2002) 168:6159–64. doi: 10.4049/jimmunol.168.12.6159 12055228

[16] Ferraro A Socci C Stabilini A Valle A Monti P Piemonti L. Expansion of Th17 cells and functional defects in T regulatory cells are key features of the pancreatic lymph nodes in patients with type 1 diabetes. Diabetes. (2011) 60:2903–13. doi: 10.2337/db11-0090 PMC3198077 21896932

[17] Hull CM Peakman M Tree TIM. Regulatory T cell dysfunction in type 1 diabetes: what’s broken and how can we fix it? Diabetologia. (2017) 60:1839–50. doi: 10.1007/s00125-017-4377-1 PMC6448885 28770318

[18] Tang Q Henriksen KJ Bi M Finger EB Szot G Ye J. *In vitro*-expanded antigen-specific regulatory T cells suppress autoimmune diabetes. J Exp Med. (2004) 199:1455–65. doi: 10.1084/jem.20040139 PMC2211775 15184499

[19] Tarbell KV Petit L Zuo X Toy P Luo X Mqadmi A. Dendritic cell-expanded, islet-specific CD4+ CD25+ CD62L+ regulatory T cells restore normoglycemia in diabetic NOD mice. J Exp Med. (2007) 204:191–201. doi: 10.1084/jem.20061631 17210729 PMC2118426

[20] Yang SJ Singh AK Drow T Tappen T Honaker Y Barahmand-Pour-Whitman F. Pancreatic islet-specific engineered T(regs) exhibit robust antigen-specific and bystander immune suppression in type 1 diabetes models. Sci Transl Med. (2022) 14:eabn1716. doi: 10.1126/scitranslmed.abn1716 36197963

[21] Zhang L Sosinowski T Cox AR Cepeda JR Sekhar NS Hartig SM. Chimeric antigen receptor (CAR) T cells targeting a pathogenic MHC class II:peptide complex modulate the progression of autoimmune diabetes. J Autoimmun. (2019) 96:50–8. doi: 10.1016/j.jaut.2018.08.004 PMC6541442 30122420

[22] Kobayashi S Thelin MA Parrish HL Deshpande NR Lee MS Karimzadeh A. A biomimetic five-module chimeric antigen receptor ((5M)CAR) designed to target and eliminate antigen-specific T cells. Proc Natl Acad Sci U S A. (2020) 117:28950–9. doi: 10.1073/pnas.2012495117 PMC7682351 33139567

[23] Tenspolde M Zimmermann K Weber LC Hapke M Lieber M Dywicki J. Regulatory T cells engineered with a novel insulin-specific chimeric antigen receptor as a candidate immunotherapy for type 1 diabetes. J Autoimmun. (2019) 103:102289. doi: 10.1016/j.jaut.2019.05.017 31176558

[24] Spanier JA Fung V Wardell CM Alkhatib MH Chen Y Swanson LA. Tregs with an MHC class II peptide-specific chimeric antigen receptor prevent autoimmune diabetes in mice. J Clin Invest. (2023) 133:e168601. doi: 10.1172/JCI168601 37561596 PMC10503798

[25] Obarorakpor N Patel D Boyarov R Amarsaikhan N Cepeda JR Eastes D. Regulatory T cells targeting a pathogenic MHC class II: Insulin peptide epitope postpone spontaneous autoimmune diabetes. Front Immunol. (2023) 14:1207108. doi: 10.3389/fimmu.2023.1207108 37593744 PMC10428008

[26] Herold KC Bundy BN Long SA Bluestone JA DiMeglio LA Dufort MJ. Teplizumab in relatives at risk for type 1 diabetes. N Engl J Med. (2019) 381:603–13. doi: 10.1056/NEJMoa1902226. PMC6776880 31180194

[27] Herold KC Gitelman SE Gottlieb PA Knecht LA Raymond R Ramos EL. Teplizumab: A disease-modifying therapy for type 1 diabetes that preserves beta-cell function. Diabetes Care. (2023) 46:1848–56. doi: 10.2337/dc23-0675 PMC10545553 37607392

