# Supplementary material for: Regulatory CD4+ T cells redirected against pathogenic CD8+ T cells protect NOD mice from development of autoimmune diabetes
Source: Front Immunol. 2024 Sep 16;15:1463971. doi: 10.3389/fimmu.2024.1463971 (PMC11439686; doi:10.3389/fimmu.2024.1463971)
Supplement: Supplementary file 1 [file DataSheet1.pdf]

Supplementary materials

Fig. S1

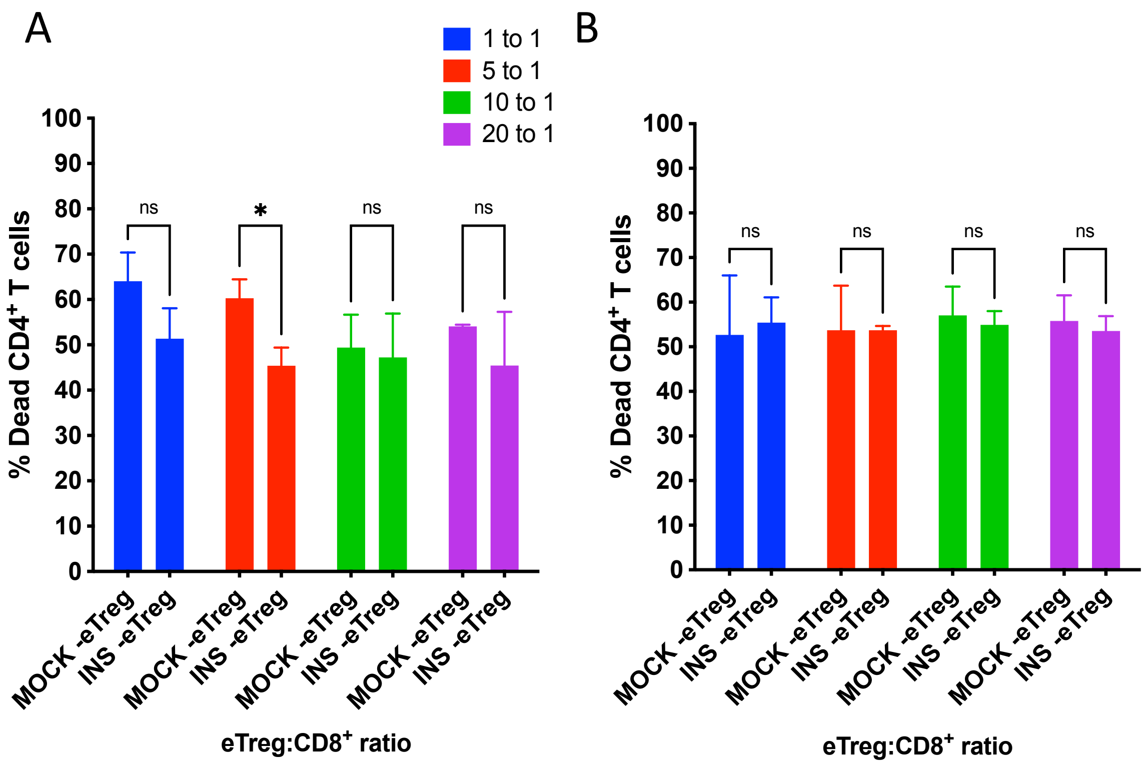

Supplementary Fig.1

Antigen–specific CD8 T cells do not cause death of INS-eTreg or IGRP-eTreg cells.

INS-eTreg or IGRP-eTreg were cultured with their cognate $CD8^+$ T cells for 24 hours at different eTreg:$CD8^+$ T cell ratios. $CD4^+$ T cell death was assessed by Live/dead viability dye. 2 experiments were carried out in duplicate. Statistical analysis performed by 2-way ANOVA. Ns – not statistically significant; $*p<0.05$

Fig. S2

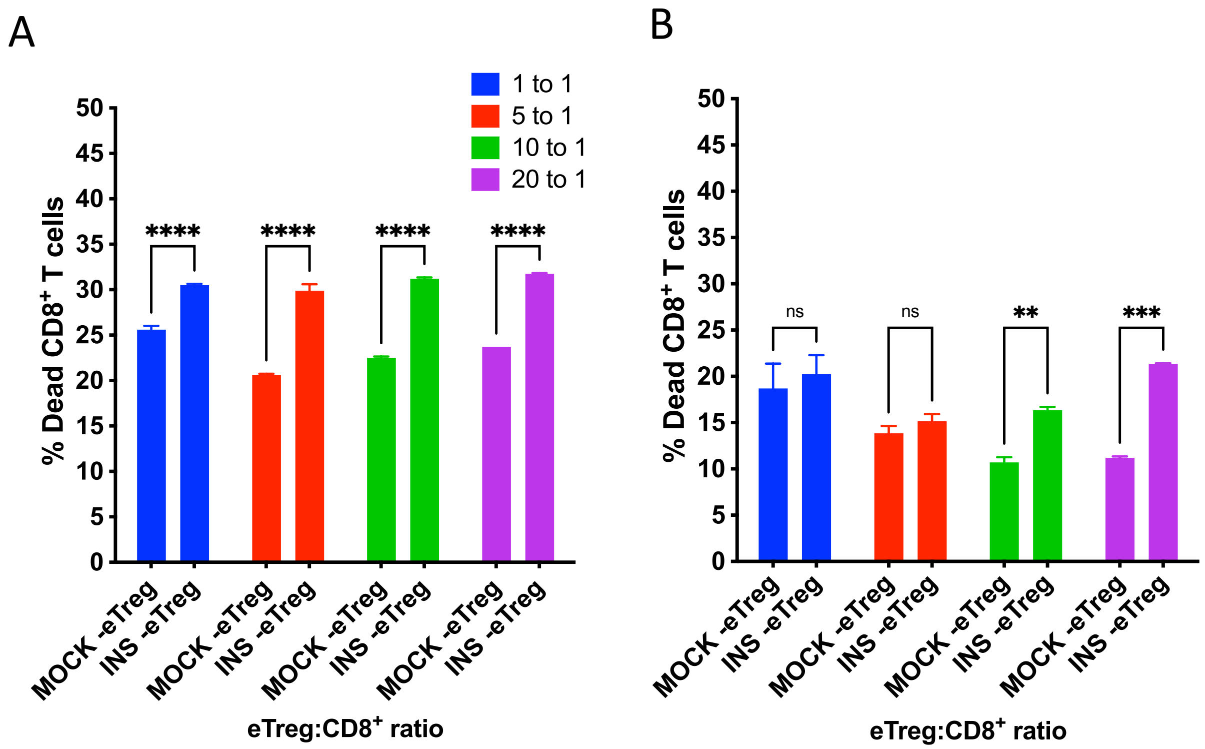

Supplementary Fig.2

Antigen–specific INS-eTreg or IGRP-eTreg CD4^{+} T cells increase death of antigen-specific CD8^{+} T cells in culture.

INS-eTreg or IGRP-eTreg were cultured with their cognate CD8^{+} T cells for 24 hours at different eTreg:CD8^{+} T cell ratios. CD8^{+} T cell death was assessed by Live/dead viability dye. 2 experiments were carried out in duplicate. Statistical analysis performed by 2-way ANOVA. ns – not statistically significant; $^{**}p<0.01$, $^{***}p<0.001$, $^{****}p<0.0001$
